# A Novel Framework with High Diagnostic Sensitivity for Lung Cancer Detection by Electronic Nose

**DOI:** 10.3390/s19235333

**Published:** 2019-12-03

**Authors:** Binchun Lu, Lidan Fu, Bo Nie, Zhiyun Peng, Hongying Liu

**Affiliations:** 1 Chongqing University-University of Cincinnati Joint Co-op Institute, Chongqing University, Chongqing 400030, China; lubu@mail.uc.edu (B.L.); fuln@mail.uc.edu (L.F.); 2 Key Laboratory of Biotechnology Science and Technology, Ministry of Education, College of Bioengineering, Chongqing University, Chongqing 400030, China; 20165686@cqu.edu.cn; 3 State Key Laboratory of Power Transmission Equipment & System Security and New Technology, Chongqing University, Chongqing 400030, China; zhiyun.peng@cqu.edu.cn

**Keywords:** lung cancer, autoencoder, ensemble pruning, electronic nose, volatile organic compounds

## Abstract

The electronic nose (e-nose) system is a newly developing detection technology for its advantages of non-invasiveness, simple operation, and low cost. However, lung cancer screening through e-nose requires effective pattern recognition frameworks. Existing frameworks rely heavily on hand-crafted features and have relatively low diagnostic sensitivity. To handle these problems, gated recurrent unit based autoencoder (GRU-AE) is adopted to automatically extract features from temporal and high-dimensional e-nose data. Moreover, we propose a novel margin and sensitivity based ordering ensemble pruning (MSEP) model for effective classification. The proposed heuristic model aims to reduce missed diagnosis rate of lung cancer patients while maintaining a high rate of overall identification. In the experiments, five state-of-the-art classification models and two popular dimensionality reduction methods were involved for comparison to demonstrate the validity of the proposed GRU-AE-MSEP framework, through 214 collected breath samples measured by e-nose. Experimental results indicated that the proposed intelligent framework achieved high sensitivity of 94.22%, accuracy of 93.55%, and specificity of 92.80%, thereby providing a new practical means for wide disease screening by e-nose in medical scenarios.

## 1. Introduction

As estimated, lung cancer has been responsible for close to 1 in 5 deaths in 2018, which remains the leading cause of cancer death [1]. According to the latest TNM 8 edition, the five-year average survival rate of stage IVA patients is 10%, and that of stage IVB patients is as low as 0% [2]. Despite the high mortality rate, early diagnosis can increase the chance of efficient treatment [3] and survival rate for lung cancer patients [4]. Radiological detection, such as computed tomography or positron-emission tomography, has enabled the lungs to be imaged for diagnosis of cancer [5]. However, these conventional detection methods are expensive and occasionally miss tumors (low sensitivity), and therefore cannot be used as widespread screening tools [6]. Moreover, radiation from medical imaging may cause adverse health effect on the human body [7]. Therefore, it is crucial to develop an effective diagnosis method for lung cancer, which is also feasible for wide screening with high sensitivity, especially for high risk patients [8].

Human volatilome analysis is a new and promising area in disease detection [9]. As a non-invasive tool for lung cancer detection [10,11], breath analysis becomes a fast-growing research field [12,13]. More than 3000 volatile organic compounds (VOCs) are found in human exhaled breath, which are directly or indirectly related to internal biochemical processes in the human body [14]. Breath print, interpreted as VOCs inside exhaled breath [15], can be analyzed by different instruments such as gas chromatography in combination with mass spectrometry (GC-MS), proton-transfer-reaction mass spectrometry, ion mobility spectrometry, and electronic nose (e-nose) [16]. E-noses are sensor arrays that consist of non-selective chemical sensors and each sensor is sensitive to a large number of VOCs with different sensitivity [17]. E-noses have been widely used in food analysis [18], environment control [19], and disease diagnosis [20]. As a promising non-invasive detection device, e-noses can identify different diseases such as lung cancer [21], prostate cancer [22], urinary tract infections [23], urinary pathogens [24], and gut bacterial populations [25]. Different from those expensive, time-consuming and complicated analysis methods by compounds identification, e-nose is popular as a simple, inexpensive, and portable sensing technology in lung cancer detection, but it relies heavily on computer analysis [26].

Although new computer-assisted diagnosis (CAD) methods emerge continuously and rapidly, effective algorithms of analyzing e-nose data for lung cancer remain far from perfection. Since e-nose cannot directly distinguish between specific VOCs [26], in addition to effective sample acquisition, another key procedure is the follow-up signal processing by using computer methods. In e-nose detection, feature extraction and classification are two basic and essential steps. Feature extraction methods are applied for analyzing high-dimensional signal data, which is the prerequisite for subsequent detection. Classification models aim to study the difference of the sensor features under different physiological conditions to achieve final diagnosis. There are many pattern recognition frameworks in diagnosing diseases by e-nose, as shown in Table 1. It can be concluded that data processing is a pivotal step to develop effective e-nose diagnosis system, which requires further improvement.

As an unsupervised learning method, autoencoder demonstrated strength in extracting relevant information from high-dimensional signal data [35]. Meanwhile, gated recurrent unit (GRU) [36] has been shown to be one of the state-of-the-art architectures in extracting temporal features. Compared with long short-term memory (LSTM) [37], GRU has no cell state and directly employs hidden state for the transmission of signal information, thus possessing rapid training time. Thus far, deep learning algorithms have only been sparsely applied for feature extraction on e-nose data. Gated recurrent unit based autoencoder (GRU-AE) integrates GRU with the autoencoder, which leverages GRU cells to discover the dependency and temporality among multi-dimensional time series signal [38]. By introducing GRU-AE into the field of e-nose analysis for lung cancer detection, the effort to manually engineer complex features is minimized, which tremendously simplifies data processing procedures for e-noses.

As for classification models, ensemble learning has been a popular and desirable learning paradigm for the analysis of e-nose data [39]. The basic idea of ensemble learning is to build multiple component learners whose predictions are aggregated with the aim of outperforming the constituent members [40]. Typically, ensemble learning algorithms consist of two stages: the production of diverse base learners and their combination [41]. High precision and diversity are two key requirements for individual learners to guarantee the performance of the final ensemble [42]. However, combining all the individual learners requires massive storage and computing resources. Even worse, the larger size of the ensemble model cannot constantly guarantee the better performance [43]. For these reasons, ensemble pruning has arisen as an intermediate stage prior to combination, which is also termed as ensemble thinning, selective ensemble, or ensemble selection [41]. Ensemble pruning searches a good subset of base learners to form the sub-ensemble that can reduce the ensemble size and resource consumption while maintaining or even enhancing the performance of the complete ensemble. However, the complexity of finding the best sub-ensemble is an NP-complete problem [44], and therefore the optimal solution by global search is infeasible for large or even medium ensemble size [45]. Alternatively, it is more appropriate to use approximation techniques that guarantee the near-optimal sub-ensembles.

Many ensemble pruning strategies have been proposed to obtain the optimal or near-optimal sub-ensembles, which can be mainly categorized into ordering-based techniques [46,47], clustering-based techniques [48], and optimization-based techniques [43,49]. Ordering-based techniques attempt to rank individual classifiers based on the evaluation measures, and only the first few classifiers are selected in the pruned ensemble. Since the ranking mechanism tends to consume less time and storage resources, ordering-based ensemble pruning is the simplest and fastest one among all the ensemble pruning techniques, which is widely applied as CAD models with high accuracy [50].

Therefore, in this paper, a novel gated recurrent unit based autoencoder combined with margin and sensitivity based ordering ensemble pruning (GRU-AE-MSEP) framework is proposed. This framework consists of three major steps. (1) The GRU-AE is adopted to extract principal features from high-dimensional and complex signal data. (2) The compressed features are used to train classification and regression trees (CARTs). (3) MSEP is employed to order and select well-trained CARTs to form final sub-ensemble for lung cancer classification. Correspondingly, the main contributions of this study are listed as follows:

1. For the first time in the field of lung cancer screening, GRU-AE is introduced into the feature extraction of e-nose signal data. As far as we know, this fills the gap of applying deep learning methods to automatically extract principal features from temporal and high-dimensional data in the e-nose system.
2. Based on the gained insight through theoretical analysis of three other ensemble pruning measures, we design and propose a heuristic margin and sensitivity based measure (MSM) for explicitly evaluating the contribution of each component classifier, which considers both instance importance and classification sensitivity. Previous studies only focused on improving the recognition accuracy of the model. To our knowledge, this is the first time that sensitivity is introduced into ensemble pruning to meet the needs of medical fields.
3. A novel MSEP is established for lung cancer detection. The proposed ensemble pruning model contributes to increasing the survival rate by decreasing missed diagnosis of lung cancer patients while guaranteeing overall performance.
4. Compared with other state-of-the-art frameworks, we demonstrate the feasibility and effectiveness of the proposed framework on collected breath samples by e-nose and three open source datasets. Therefore, the proposed intelligent framework provides a new insight into machine learning algorithms and lung cancer detection.

The remainder of this paper is organized as follows. In Section 2, the acquisition process and pre-processing of the collected data are explained and summarized. Section 3 proposes the feature extraction method of GRU-AE and classification models based on ensemble pruning techniques. In Section 4, the performance of the proposed framework is tested and further validated by comparison with other algorithms. Discussion is shown in Section 5. Finally, Section 6 draws some conclusions of this study.

## 2. Materials

### 2.1. Data Collection

In this study, a total of 214 breath samples were collected from 98 patients with lung cancer and 116 heathy controls. Lung cancer patients were from the in-patient department of the Chongqing Cancer Hospital and Chongqing Red Cross Hospital. Healthy volunteers were doctors and nurses in the Chongqing Cancer Hospital and researchers from Chongqing University. All participants confirmed that they had no metabolic comorbidities and none of the patients had their tumors removed. After a detailed introduction of the purpose and plan of this experiment, all subjects gave their informed consent for inclusion before participating in the study. This study was conducted in accordance with the Declaration of Helsinki. Protocols including any relevant details of this study were carried out in accordance with the relevant guidelines and approved by Medical Ethics Committee of Chongqing Cancer Hospital as well as Medical Ethics Committee of Chongqing Red Cross Hospital. Table 2 provides the overall information of the volunteers participating in this study.

The breath collection process was standardized and based on a validated study published previously [51]. In brief, during the process of collection, all the volunteers blew the gas into the bag after deep breathing. To reduce the interference in the breath composition on account of different lifestyles, different variables were controlled such as the time interval, temperature, oral hygiene, etc. Sampling experiments were conducted in well-ventilated rooms to avoid interference by other odors. The detection process was carried out immediately after sample acquisition. The data used for classification were response signals from 13 sensors, including TGS2620, TGS2602, TGS2600, TGS826, TGS822, TGS8669, WSP2110, NAP-55A, MR516, ME3-C7H8, CO-B4, a temperature sensor, and a humidity sensor.

### 2.2. Breath Preprocessing

The miniature e-nose system in this study is composed of lower computer system, upper computer software, and data processing system. The lower system consists of gas chamber, sensor array, and signal processing circuit. The upper computer stores information of the users and detection data in MySQL (Oracle, CA, USA) relational management database. The upper computer and the lower computer of the e-nose system are combined to obtain the data of the samples and then store them to the database.

**Figure 1:**
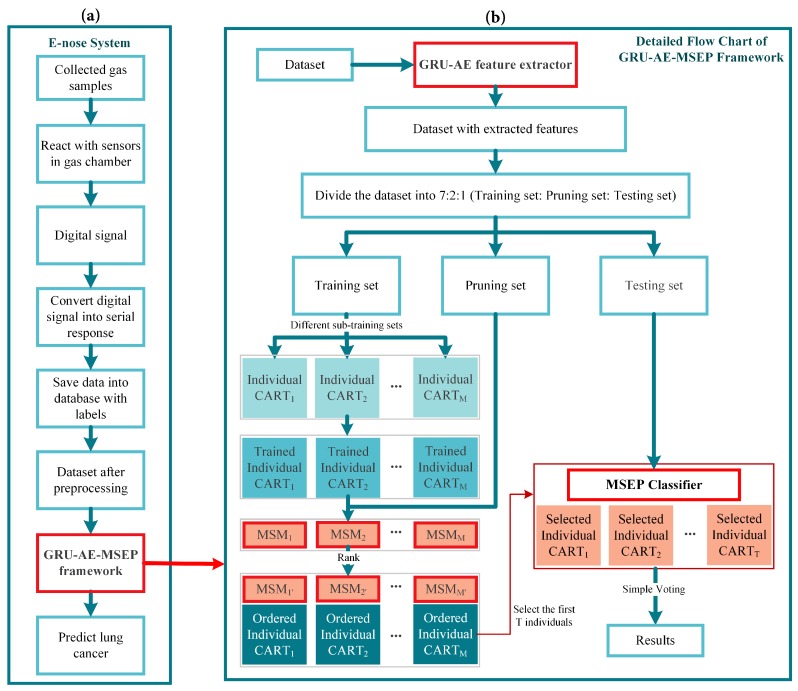
Overall scheme of the research: (**a**) the computer-assisted diagnosis (CAD) system of electronic nose (e-nose); and (**b**) the proposed framework.

The overall scheme of e-nose detection system consists of the eight major steps shown in Figure 1a. Firstly, breath samples of volunteers were gathered, and then samples containing VOCs were sent into the gas chamber one by one. The sample gas diffused and eventually reached a uniform distribution. After reacting with the sensor arrays in the gas chamber, the machine outputted electrical signals. In the third step, electrical signals were amplified, filtered, and converted to digital signals. Then, digital signals were uploaded to the upper computer system via serial asynchronous communication. The upper computer system displayed the real-time response of the sensors, and saved data to the local database. The sixth step is the pre-processing of saved sensor data, including baseline processing, filtering and data standardization. Thereinto, baseline processing was used to achieve purposes such as drift compensation and contrast enhancement. As for the filtering of sensor signals, the wavelet filtering was applied owing to its fast computation capability and wide adaptability. The reaction time for each sensor was 90 s and each sensor collected 675 points in this time interval. Therefore, every sample datum had the dimensionality of 8775, i.e., 13 sensors multiplied by 675 time steps. Ultimately, the pre-processed signal data were analyzed through the pattern recognition framework.

## 3. Methodology

The algorithms used in the proposed framework are explained and interpreted below. The pipeline of the whole detection framework is shown in Figure 1b. Firstly, pre-processed data were inputted into the GRU-AE-MSEP framework. GRU-AE was then trained to extract principal features from each sample. After being trained on the training set, CARTs were ordered and selected by the MSEP on the pruning set step by step. Finally, the selected classifiers formed the pruned ensemble to make predictions and obtained classification results through simple voting on the testing set.

### 3.1. Feature Extraction

**Figure 2:**
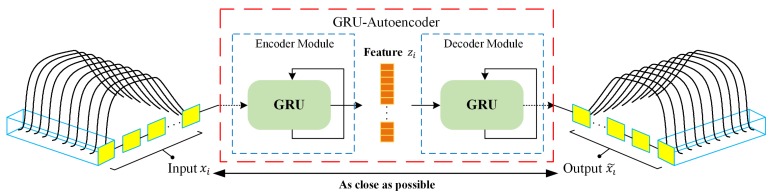
Feature extraction process of the gated recurrent unit based autoencoder (GRU-AE).

In this study, GRU-AE was applied to form elaborate feature representation to achieve effective classification subsequently. The schematic diagram of GRU-AE for feature extraction is illustrated in Figure 2. Generally, encoder module and decoder module are two fundamental components in the autoencoder. The encoder transforms the high-dimensional data $x_{i}$, which consist of multichannel signals, into a compressed representation $z_{i}$. The decoder module then implements the conversion from compressed features to original high-dimensional data, denoted as output ${\tilde{x}}_{i}$. The autoencoder attempts to minimize the reconstruction error in Equation (1), which is defined as the difference between the $x_{i}$ and ${\tilde{x}}_{i}$, where *D* is the dimensionality of the input. Finally, $z_{i}$ can be regarded as a valid representation of the input signal.

(1) $$MSE=\frac{1}{D}\sum_{m=1}^{D}{\left(x_{i}^{m}-{\tilde{x}}_{i}^{m}\right)}^{2}$$

GRU-AE model integrates GRU cells with the autoencoder, which means the encoding and decoding processes are implemented by GRU [36]. In GRU-AE, GRU cells are leveraged to discover the dependency and correlations among multi-dimensional time series signal. As  shown in Figure 2, GRU can process responses of multiple sensors simultaneously at each time step, and then generates sequence information in the encoder module. After training GRU-AE by back propagation algorithm, low-dimensional $z_{i}$ serves as temporal features extracted by the autoencoder and can appropriately represent the input signal $x_{i}$. More detailed description and principle of GRU-AE can be found in [38].

### 3.2. Ensemble Pruning for Classification

In this section, the margin theory of ensemble method is interpreted and applied to investigate the relationship between samples and classifiers. Then, the advantages and shortcomings of three different ensemble pruning measures are analyzed and evaluated. By analysis and comparison, we propose a heuristic measure based on margin theory to assess the importance of each individual classifier, which can effectively rank and prune the base classifiers to construct a near-optimal sub-ensemble.

First, all the notations used in this section are introduced, which helps to comprehend the measures mentioned in this paper. Let *D* = {($x_{i}$, $y_{i}$)|*i* = 1, 2, …, *N*} be the total dataset constituted by each sample $x_{i}$ with the corresponding label $y_{i}$∈ {0, 1}, which can be divided into $D_{Tr}$ with size of $N_{Tr}$ for training, $D_{Pr}$ with size of $N_{Pr}$ for pruning, and $D_{Te}$ with size of $N_{Te}$ for testing. The base classifier is denoted as $h_{i}$, which is used to compose the original ensemble set *H* with *M* classifiers, and ensemble pruning set *S* with *T* classifiers. Suppose *I* is the discriminant equation where *I*(*true*) = 1 and *I*(*false*) = 0.

#### 3.2.1. Margin Theory

The margin theory was originally proposed to analyze the upper bound of generalization error for ensemble methods with voting classification rules [52]. To further explain the correctness of the margin theory, *k*th margin bound was proposed to narrow the upper bound of generalization error with respect to margin distribution [53,54]. From the margin theory, it can be concluded that the larger is the margin over the training samples, the better is the generalization performance of the ensemble model on the testing set. Consider a binary classification problem, whose prediction is the result of majority voting. The margin of the sample $x_{i}$ is defined as Equation (2), which is a number in the range of [−1, 1].

(2) $$margin\left(x_{i}\right)=\frac{\sum_{j=1}^{M}(I\left(h_{j}\left(x_{i}\right)=y_{i}\right)-\left(I\left(h_{j}\left(x_{i}\right)\neq y_{i}\right)\right)}{M}$$

Margin is a measure of the confidence for ensemble prediction [52]. From Equation (2), the larger positive (or negative) value of margin indicates the more confident correct (or incorrect) prediction. Since better generalization performance can be achieved by larger margin on the whole training samples, the individual classifiers that make correct predictions are more important than those that make incorrect predictions. Intuitively, the larger is the negative margin of the sample, the more important are the base classifiers who can correctly classify it, since such classifiers have the potential to guide the ensemble to make the correct prediction. Based on those insights, margin-based measures can be applied to selecting appropriate individual classifiers.

#### 3.2.2. Reviews and Analyses of Three Ensemble Pruning Measures

Before introducing proposed margin-based ensemble pruning algorithm, we first illustrate three different measures for ensemble pruning as guidance: simultaneous diversity and accuracy measure for ensemble pruning (SDAcc) [55], margin and diversity based ordering ensemble pruning (MDEP) [47], and unsupervised margin based ordering ensemble pruning (UMEP) [46]. For clarity and coherence, without altering the original meaning of the above three methods, the following formulas are based on the notations defined in this study.

(3) $$SDA\mathrm{cc}\left(h,\;S\right)=\sum_{\left(x_{i},\;y_{i}\right)\in D_{Pr}}\begin{array}{c} (I\left(e_{10}\left(h,\;S,\;x_{i},\;y_{i}\right)\right)NF_{i}^{S}+I\left(e_{11}\left(h,\;S,\;x_{i},\;y_{i}\right)\right)NF_{i}^{S}\\ -I\left(e_{01}\left(h,\;S,\;x_{i},\;y_{i}\right)\right)NF_{i}^{S}-I\left(e_{00}\left(h,\;S,\;x_{i},\;y_{i}\right)\right)NT_{i}^{S}) \end{array}$$

(4) $$\begin{array}{c} e_{00}\left(h,S,x_{i},y_{i}\right):h\left(x_{i}\right)\neq y_{i}\&S\left(x_{i}\right)\neq y_{i}\\ e_{01}\left(h,S,x_{i},y_{i}\right):h\left(x_{i}\right)\neq y_{i}\&S\left(x_{i}\right)=y_{i}\\ e_{10}\left(h,S,x_{i},y_{i}\right):h\left(x_{i}\right)=y_{i}\&S\left(x_{i}\right)\neq y_{i}\\ e_{11}\left(h,S,x_{i},y_{i}\right):h\left(x_{i}\right)=y_{i}\&S\left(x_{i}\right)=y_{i} \end{array}$$

(5) $$NT_{i}^{S}=\frac{\sum_{j=1}^{T}I(h_{j}\left(x_{i}\right)=y_{i})}{T}$$

To improve the error-correction ability and ensure the effectiveness of the pruned ensemble, both the accuracy and diversity of an individual classifier should be considered [55]. SDAcc shown in Equation (3) proposes a measure to combine different weights for four events, which can primarily care about accuracy and diversity of the sub-ensemble. Four events in the measure are defined in Equation (4), where *h* is an individual classifier to make predictions in the pruned ensemble *S*. In Equation (5), $NT_{i}^{S}$ denotes the correct classification ratio on the pruning dataset $D_{Pr}$, and $NF_{i}^{S}$ is equal to $1-NT_{i}^{S}$. The measure in SDAcc gives marks for classifiers with correct prediction and deducts corresponding marks for incorrect classifiers. $e_{10}$ and $e_{11}$ indicate two cases where the base classifier make the correct decision, and the base classifier can be rewarded with different high marks, i.e., $NF_{i}^{S}>0.5$ in $e_{10}$ and $NF_{i}^{S}<0.5$ in $e_{11}$. In event $e_{00}$, since the results of the base classifier and the ensemble are the same, the base classifier lacks diversity. At the same time, the result of the base classifier is wrong, which makes it lack accuracy. Classifiers in $e_{00}$ have both low accuracy and diversity, and therefore should be deducted more marks than that in $e_{01}$. Through SDAcc, the candidates with high accuracy and diversity can be selected for the final sub-ensemble. However, this measure was designed for the optimization process in greedy ensemble pruning, thus possessing higher complexity than the ordering-based ensemble pruning. Moreover, the incorrect classifiers in the case $e_{01}$ and $e_{00}$ have overlapped mark intervals, which means two samples with different importance could be considered equally important. For instance, a base classifier makes wrong prediction on the sample with 80 correct votes and 20 incorrect votes (belongs to $e_{01}$), while the other base classifier incorrectly classifies a sample with 80 incorrect votes and 20 correct votes (belongs to $e_{00}$). However, the marks for the incorrect classifiers in above two different events are all −0.2. Hence, it is hard to distinguish the importance of each classifier by its mark values in SDAcc. Moreover, diversity cannot guarantee the generalization capacity of the final pruned ensemble [56]. The following two ensemble pruning measures use margin theory to evaluate the importance of base classifiers in a relatively reliable manner.

(6) $$MDM\left(h,H\right)=\sum_{x_{i}\in D_{Pr}}\left[I\left(h\left(x_{i}\right)=y_{i}\right)\left(\alpha f_{m}\left(x_{i}\right)+\left(1-\alpha \right)f_{d}\left(h,x_{i}\right)\right)\right]$$

(7) $$f_{m}\left(x_{i}\right)=-\log\left(\left|margin(x_{i})\right|\right)$$

(8) $$f_{d}\left(h,x_{i}\right)=-\log\left(\frac{\sum_{j=1}^{M}\left(I\left(h\left(x_{i}\right)=y_{i}\right)\right)}{M}\right)$$

MDEP is an ordering-based ensemble pruning model which relies on the margin and diversity based measure (MDM) [47]. Since large margin can guarantee high generalization capacity, base classifiers that have the ability to increase instance margin should be first considered. The article states that the importance of each sample increases as the absolute margin value decreases, therefore the logarithmic function is used to reveal such tendency. MDM shown in Equation (6) linearly combines the margin measure shown in Equation (7) and the diversity measure shown in Equation (8) with an adjustable parameter α. However, MDM deliberately favors the candidates that can make correct decisions on samples with low (positive or negative) margin. The samples that have large negative and large positive margin are considered equally in MDM, which both have little importance. However, in our opinion, since every sample is unique and considerable, hard samples should not be totally neglected, especially in the medical scenarios. Those difficult samples with large negative margin must be valued, which is the key to further improving the accuracy and sensitivity of the ensemble pruning model. Moreover, classifiers that can correctly classify the samples with most incorrect votes (margin<0) should be more important. For instance, a sample $x_{p}$ has 55 incorrect votes and 45 correct votes ($margin(x_{p})<0$), while the other sample $x_{q}$ has 45 incorrect votes and 55 correct votes ($margin(x_{q})>0$). Classifiers that can correctly classify $x_{p}$ should be more important than classifiers that can correctly classify $x_{q}$. However, in MDEP, the above two cases are of equal importance.

(9) $$UMEP(h,H)=\frac{1}{N_{Pr}}\sum_{\left(x_{i},y_{i}\right)\in D_{Pr}}-\log(margin\left(x_{i}\right))$$

The UMEP [46] model highlights the main impact of low margin samples on the performance of pruning tasks. The logarithmic function was also applied to represent the inverse relation between the importance of the classifier and the margin of samples, as  shown in Equation (9). The lower is the margin of sample $x_{i}$, the higher is the information quantity in $x_{i}$, and therefore the more significant is the classifier that makes correct decision on $x_{i}$. The article emphasizes that the margin-based ordering classifiers are less likely to make coincident errors, and therefore sufficient diversity can be ensured compared to other ordering-based methods with the same complexity. Nevertheless, the logarithmic function can only deal with positive values and samples with negative margin are entirely neglected. As mentioned above, those samples misclassified by most classifiers (margin<0) should be taken seriously rather than discarded.

#### 3.2.3. Proposed Margin and Sensitivity Based Measure

Different from the three measures for ensemble pruning mentioned above, i.e., SDAcc, MDEP, and UMEP, this study has distinctive standpoint about margin distribution on different cases in medical scenarios. We propose herein a novel measure called the margin and sensitivity based measure (MSM) for base classifiers as:

(10) $$MSM=\frac{1}{N_{Pr}}\sum_{\left(x_{i},y_{i}\right)\in D_{Pr}}I\left(h\left(x_{i}\right)=y_{i}\right)\cdot I\left(margin\left(x_{i}\right)>\theta \right)\cdot e^{y_{i}\cdot NF_{i}^{H}}\cdot e^{-margin(x_{i})}$$

(11) $$NF_{i}^{H}=\frac{\sum_{j=1}^{M}I(h_{j}\left(x_{i}\right)\neq y_{i})}{M}$$

The motivation of designing MSM is to take into account the importance and difference between candidate classifiers, and, simultaneously, to consider the classification sensitivity while maintaining overall performance. To obtain a reasonable evaluation based on margin theory, the fourth term, i.e., $e^{-margin(x_{i})}$, is invented for the following three reasons: (1) Instead of logarithmic function, which is discontinuous on the whole interval of margin ([−1, 1]) and has infinite values, exponential function is utilized to depict the importance of different base classifiers. (2) The fourth term covers the interval [−1, 0], which means that very hard samples are considered as well. Moreover, samples misclassified by most base classifiers are given more attention. Therefore, the importance of the sample can be reflected precisely in each situation. (3) For samples that have more incorrect votes (the smaller margin), the classifiers that can correctly classify them deserve higher marks, while for samples with more correct votes (the larger margin), the correct classifiers should get lower marks. Therefore, by following the three above rules, the fourth term is monotonically decreasing, and sufficient diversity of the sub-ensemble can be achieved by distinguishable marking mechanism for different situations.

For serious diseases detection such as cancer, the rate of missed diagnosis should be reduced to increase the cure possibility, which motivates the creation of the third term in the MSM. $e^{y_{i}\cdot NF_{i}^{H}}$, referred to as the bonus term, aims to lower the rate of false negative identification, and the definition of $NF_{i}^{H}$ is shown in Equation (11). Only classifiers that can correctly detect lung cancer samples (positive cases, $y_{i}=1$) are rewarded with bonus marks, which is $e^{NF_{i}^{H}}$. In the case that more than half of the classifiers misclassify sample $x_{i}$ ($margin\left(x_{i}\right)<0\wedge NF_{i}^{H}>0.5$) and $x_{i}$ happens to be the positive sample, the correct classifiers can get much higher marks. By introducing the bonus term, classifiers that can correctly identify lung cancer samples are more likely to be favored. By modifying the bonus term, MSM can be extended to multi-class problems, allowing the classifiers that successfully distinguish the most important categories to obtain additional marks. Therefore, the bonus term makes MSM more competent to increase the sensitivity of the pruning model.

The first term, i.e., $I(h\left(x_{i}\right)=y_{i})$, determines that only the correct classifiers can earn marks. The second term, i.e., $I(margin\left(x_{i}\right)>\theta )$, called threshold, is created specially to eliminate abnormal samples. In extreme cases, if all classifiers are wrong, except one or two classifiers, then the sample is very likely to be abnormal and should be ignored. Therefore, the interval of θ is [−1, 0], which is a parameter to reduce adverse impact by outliers and elusive samples.

In contrast to SDAcc, MDEP, and UMEP, MSM aims to improve the classification sensitivity under the circumstance of maintaining high accuracy and specificity, and therefore can be widely used in the diagnosis of cancer and other serious diseases. Compared with SDAcc, MSM defines a more rational evaluation for different situations through margin theory, which can consider the difference and generalization ability simultaneously. The  marking mechanism of SDAcc linearly depends on the voting results, i.e., $NF_{i}^{S}$ and $NT_{i}^{S}$. However, the  marking mechanism of MSM has varying slopes according to the importance of samples. Classifiers that can correctly predict samples with smaller margin have greater tendency to obtain marks. Additionally, instead of abandoning hard samples in MDEP and UMEP, MSM attempts to classify samples as correctly as possible, especially the difficult ones, and therefore can further boost the performance of the ensemble pruning. Furthermore, the proposed heuristic ensemble pruning measure based on margin theory is demonstrated and verified by reasonable and exhaustive experiments in Section 4.

**Algorithm 1** Algorithm of margin and sensitivity based ordering ensemble pruning (MSEP)
 **Input:**training set $D_{Tr}$, pruning set $D_{Pr}$, sample (*x*, *y*), size of training set $N_{Tr}$, size of pruning set $N_{Pr}$, base classifier *h*, initial ensemble *H*, size of initial ensemble *M*, size of final pruned ensemble *T*, parameter θ **Output:**The final sub-ensemble *S*
1:Initialize *S* = ϕ , KL is an empty mark list;2:// Train base classifiers;3:**for** each $h_{j}\in H$ **do**4:    Extract ($x_{i}$, $y_{i}$) ∈ $D_{Tr}$ with replacement as $E_{Tr}$ with size of 30% × $N_{Tr}$;5:    Train $h_{j}$ with $E_{Tr}$;6:
**end for**
7:// Pruning procedures;8:**for** each $h_{j}\in H$ **do**9:    MSM=0;10:    **for** each $x_{i}\in D_{Pr}$ **do**11:        $margin\left(x_{i}\right)=\frac{\sum_{j=1}^{M}(I\left(h_{j}\left(x_{i}\right)=y_{i}\right)-\left(I\left(h_{j}\left(x_{i}\right)\neq y_{i}\right)\right)}{M}$;     ▹ refer to Equation (2)12:        **if** $h_{j}\left(x_{i}\right)=y_{i}$ **&&** $margin(x_{i})>\theta$ **then**       ▹ refer to Equation (10)13:           $NF_{i}^{H}=\frac{\sum_{j=1}^{M}I(h_{j}\left(x_{i}\right)\neq y_{i})}{M}$;             ▹ refer to Equation (11)14:           $MSM=MSM+e^{y_{i}\cdot NF_{i}^{H}}\cdot e^{-margin(x_{i})}$;      ▹ refer to Equation (10)15:        **end if**16:    **end for**17:    $MSM=\frac{MSM}{N_{Pr}}$;                  ▹ refer to Equation (10)18:    Append pair $\left(h_{j},MSM\right)$ to KL;19:
**end for**
20:Rank KL in decreasing order based on MSM;21:**return** the top-*T* classifiers in KL as *S*;


#### 3.2.4. Margin and Sensitivity Based Ordering Ensemble Pruning

In this study, MSM is applied to create an ordering-based ensemble pruning, i.e., MSEP. Sampling with replacement is employed to produce diversity on the sub-training dataset. Overproduced CARTs are trained as base classifiers by using sub-training sets from the above sampling. Finally, the trained CARTs are ranked and selected by MSM from the original ensemble to form the sub-optimal ensemble. The algorithm of MSEP is provided in Algorithm 1, which can be implemented by the following five steps:

1. MSEP starts by generating *M* CARTs from extracted sub-training set $E_{Tr}$ with replacement. Each sub-training set is different with size of 30% of the total training set $D_{Tr}$, and therefore each well-trained CART is unique and diverse.
2. Classify each sample in the pruning set $D_{Pr}$ by each well-trained CART and compute the margin value of each sample through Equation (2).
3. Only classifiers that properly predict the samples with margin larger than the threshold θ can get positive marks through Equation (10). Then, all *M* CARTs are sorted by corresponding marks into ordered sequence $h_{1}^{R},h_{2}^{R},\ldots ,h_{M}^{R}$ such that $MSM\left(h_{m}^{R}\right)>MSM\left(h_{m+1}^{R}\right)$, m≤(M−1).
4. Select the first *T* ordered CARTs to compose a pruned ensemble *S* to achieve the best overall performance including accuracy, sensitivity, and specificity.
5. Evaluate the sub-ensemble *S* over the testing set $D_{Te}$ by required metrics.

## 4. Results

### 4.1. Evaluation Metrics

The accuracy (Acc) of the classification is the proportion of correctly classified samples to the total number of samples. The classification accuracy defined in Equation (12) measures the universal classification results. TN is the number of true predictions for healthy samples; FN is the number of false predictions for healthy samples; TP is the number of true predictions for lung cancer samples; and FP is the number of false predictions for lung cancer samples.

(12) $$Acc=\frac{TP+TN}{TP+FP+TN+FN}$$

Sensitivity measures the proportion of real lung cancer patients who are correctly classified and defined as Equation (13). Instead, specificity, defined in Equation (14), measures the proportion of real healthy people who are correctly predicted. High sensitivity indicates low rate of missed diagnosis, i.e., few lung cancer patients are classified as healthy individuals, which is particularly vital for lung cancer detection. High specificity indicates low rate of misdiagnosis, i.e., few healthy individuals are deemed as lung cancer patients.

(13) $$Sen=\frac{TP}{TP+FN}$$

(14) $$Spe=\frac{TN}{FP+TN}$$

### 4.2. Experimental Methodology

In this study, the main purpose was to verify the proposed framework and explore the effect of different pruning measures on the Acc, Sen, Spe, and area under the curve (AUC) in lung cancer classification, especially on the Sen. Since the size of the ensemble should be an odd number in binary classification to avoid tie situation where every class has equal votes, the size of original ensemble set, i.e., *M*, was set to be 101 and the size of pruning set, i.e., *T*, was set to be 11 (about 10% of the original ensemble size). In the experiment, we divided the dataset into 7:2:1 for training, pruning, and testing. All experimental results were obtained by 50-fold cross-validation. The program was carried out by Python 3.6.5 and Keras 2.2.4 on Windows 10 Operating System with Intel (R) Core (TM) ( Palo Alto, CA, USA) i7-7700HQ CPU @ 2.80 GHz and 8 GB RAM.

Comparative experiments were conducted on three feature extraction methods combined with seven different classification models. In the field of e-nose system for lung cancer screening, deep learning methods have been sparsely applied to extract features. Principal component analysis (PCA) [57] and kernel principal component analysis (KPCA) [58] are the most commonly used feature extraction methods in this field. Therefore, these two dimensionality reduction methods were adopted for comparison with GRU-AE introduced in this study. As for classification, seven different models, i.e., MSEP, MEP, MDEP, UMEP, SDAcc, complete ensemble, and adaboost [59], were tested and compared. The variant from proposed method, i.e., MEP, is MSEP without the bonus term. Complete ensemble and adaboost are two widely used and successful models in machine learning. Grid-search method combined with cross-validation was employed to optimize parameters of different methods over a given parameter grid, which is shown in Table A1. The result tables in the following sections demonstrate the results of different methods under the optimal parameters. Additionally, besides examining on the collected dataset, three open source datasets were applied to further validate the proposed framework.

### 4.3. Experiments on the Lung Cancer Dataset

Firstly, binary classification experiments were carried out on the collected samples, and the proposed method was compared with other frameworks to test its performance. This part was to verify that the portable e-nose combined with the proposed framework can properly and effectively differentiate between lung cancer and healthy controls. In the second part, we investigated additional categories: (i) clinical stages; (ii) lung cancer versus chronic obstructive pulmonary disease (COPD); and (iii) smoking history by GRU-AE-MSEP to make this study more exhaustive.

#### 4.3.1. Lung Cancer versus Healthy Controls

**Figure 3:**
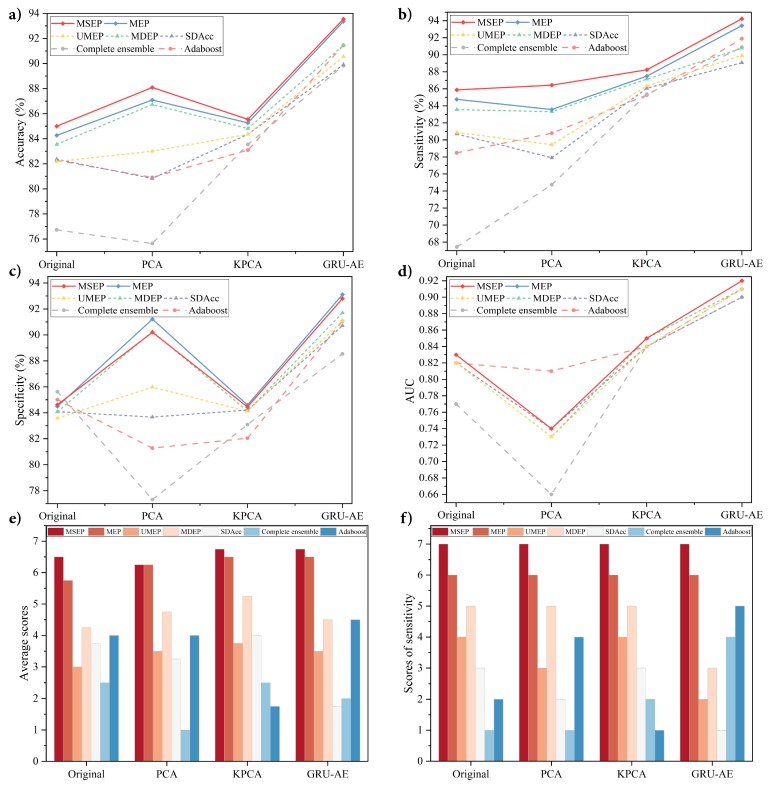
Performance comparison on the lung cancer dataset: (**a**) comparison of accuracy; (**b**) comparison of sensitivity; (**c**) comparison of specificity; (**d**) comparison of area under the curve; (**e**) average scores of seven models with original dataset and three dimensionality reduction methods; and (**f**) sensitivity scores of seven models with original dataset and three dimensionality reduction methods.

In the experiment on the collected data for binary classification, a total of 214 samples composed by 98 lung cancer patients and 116 healthy controls were utilized. Seven classification models combined with three different dimensionality reduction methods were conducted and documented. The mean values and standard deviation (std) of all the metrics obtained by 50-fold cross-validation are shown in Table 3. Figure 3 presents the comparison between different frameworks. The extensive search process of θ in MSEP is shown in Table A2.

The results shows that, among all the methods, the proposed GRU-AE-MSEP framework achieved three highest metrics, i.e., Acc of 93.55%, Sen of 94.22%, and AUC of 0.92. On the original high-dimensional dataset, MSEP achieved the highest Acc, Sen, and AUC, while complete ensemble obtained the highest Spe. As for data reduced by PCA, MSEP obtained the highest Acc and Sen. The highest Spe was obtained by MEP, and adaboost achieved the highest AUC with the largest std. On the dataset after feature extraction based on KPCA and GRU-AE, MSEP achieved the highest Acc, Sen, and AUC with small std, and MEP demonstrated the highest Spe.

To analyze the effectiveness of each feature extraction method, we compared those methods under the same classification models. Through experimental results, classification models based on GRU-AE consistently demonstrated better performance than those based on PCA and KPCA. Figure 3a–d exhibits stable ascending trend of the classification performance based on GRU-AE in every metric. In Figure 3b,c, PCA and KPCA are substantially unstable since they fail to improve the metric of all models simultaneously and the improvements are relatively small or even negative. As for the analysis of different classification models, each of them was compared under the same feature extraction methods, as shown in Figure 3e,f. In fact, when comparing two or more algorithms, a more reasonable way is to compare ranks or average ranks of different models on the same dataset [47]. Therefore, we defined a scoring rule, where the model with the highest metric gets seven points (there are seven classification models in total), the second ranked model gets six points, and so on. The scores in Figure 3e refer to the average scores on four metrics (Acc, Sen, Spe, and AUC) in four groups (Original, PCA, KPCA, and GRU-AE). For instance, MSEP combined with GRU-AE ranked the first in Acc (93.55%), Sen (94.22%), and AUC (0.92) while ranked the second in Spe (92.80%). Therefore, MSEP obtained seven, seven, six, and seven scores, correspondingly, and the average score of MSEP based on GRU-AE was 6.75. Through our scoring rule, Figure 3e directly and clearly shows the overall performance of each framework. Through the results, the proposed MSEP achieved the best overall performance among four groups. MEP and MSEP tied for the first place in the dataset based on PCA, and MEP ranked second in the other three groups. The overall performance of UMEP, SDAcc, complete ensemble, and adaboost fluctuated greatly in different groups. Figure 3f demonstrates scores of each model on sensitivity in four groups. The results illustrated that MSEP consistently achieved the highest sensitivity in different groups, while MEP had the second-best sensitivity. The sensitivity performance of other methods varied greatly among different groups.

#### 4.3.2. Various Categories Analyzed by the Proposed Framework

GRU-AE-MSEP (without the bonus term) was tested on the collected data for three classifications: (i) clinical stages; (ii) lung cancer versus COPD; and (iii) smoking history. The mean values of all the metrics were obtained by 50-fold cross-validation.

To verify whether the e-nose system had recognition effect on the staging of lung cancer, the proposed framework was tested with samples at different clinical stages. In this study, a total of 98 lung cancer samples (2 stage I, 6 stage II, 44 stage III, and 46 stage IV) were collected. Since there were only two samples for stage I, three sets of samples were employed (6 stage II, 44 stage III, and 46 stage IV) during the experiment. The results are shown in Table 4.

Lung cancer and COPD were also studied to make this research more convincing and complete. Among them, COPD patients were in-patients of Chongqing Red Cross Hospital, none of whom had lung cancer or suspected lung cancer. To avoid the influence of smoking factors on the experiment, COPD patients were all non-smoking samples. In total, 96 samples were selected, including 35 healthy non-smokers (had no lung diseases), 33 lung cancer patients (had no other lung diseases), and 28 COPD patients. The results are shown in Table 5.

In addition, a preliminary study was conducted on high-risk groups of lung cancer. In this experiment, healthy people who have been smoking for 30 years or more (1 pack or more per day) were selected as subjects. We excluded samples of long-term smokers with interfering factors such as lung diseases. Finally, 95 samples were selected, including 30 lung cancer patients (15 smokers and 15 non-smokers), 30 healthy long-term smokers, and 35 healthy non-smokers (had no smoking history). The results are shown in Table 6.

### 4.4. Experiment on Validation Datasets

In general, different datasets with sufficient size are required to test a new framework, and only convincing results can prove its stability and generalization ability. However, due to the difficulty in acquisition process, the size of VOCs dataset is relatively small, as shown in Table 1. Worse still, in terms of disease detection, there are few publicly available e-nose datasets, let alone e-nose data for lung cancer. Since the nature of e-nose response is high-dimensional and temporal data collected by chemical sensor array, we employed three related open source datasets with considerable amount, i.e., the Diabetes dataset [60], gas sensors for home activity monitoring dataset (GSHAM dataset) [61], and gas sensor array drift dataset at different concentrations (GSAD dataset) [62,63]. The approach to verify the proposed model on other datasets has been applied in similar studies (e.g., [64,65]).

#### 4.4.1. Description of Validation Datasets

Human urinary VOCs are used to diagnose diabetes in the Diabetes dataset [60]. High-dimensional time series data of VOCs in human urine were collected by field asymmetric ion mobility spectrometry. The dataset contains the urinary VOCs from two groups of people, including 72 patients with type II diabetes (set as positive samples) and 43 healthy volunteers (set as negative samples). GSHAM dataset contains high-dimensional time series data collected by eight gas sensors [61]. The sensors detected different objects by reacting with volatile gases and generated signals, which was an essential part of the e-nose detection system. There were 33 samples of banana, 36 samples of wine, and 31 samples of blank control group. In the binary classification experiment, we employed two classes with larger size, i.e., banana (positive samples) and wine (negative samples). The GSAD dataset was collected by 16 chemical sensors that reacted with pure gaseous substances [62,63]. There are 183 samples of ethanol, 209 samples of ethylene, 115 samples of ammonia, 138 samples of acetaldehyde, 214 samples of acetone, and 130 samples of toluene. Likewise, two groups were selected for binary classification, i.e., acetone (positive samples) and ethylene (negative samples) plus acetaldehyde (positive samples) and toluene (negative samples).

#### 4.4.2. Results on Validation Datasets

The results show that, among all the frameworks, the proposed GRU-AE-MSEP achieved highest Acc of 82.06%, Sen of 85.83%, and AUC of 0.73 on Diabetes dataset, and highest Acc of 89.71%, Sen of 92.87%, and AUC of 0.77 on GSHAM dataset. Since GSAD dataset did not contain time series data, GRU-AE was not applied. MSEP obtained highest Sen of 98.79%, and AUC of 0.98 on Acetone and Ethylene category. Meanwhile, MSEP achieved highest Acc of 98.96% and Sen of 98.74% on Acetaldehyde and Toluene category. Furthermore, the proposed framework was stable and achieved relatively small std while comparing with other methods. Detailed results are shown in Table A3 for Diabetes dataset, Table A4 for GSHAM dataset, and Table A5 for GSAD dataset.

**Figure 4:**
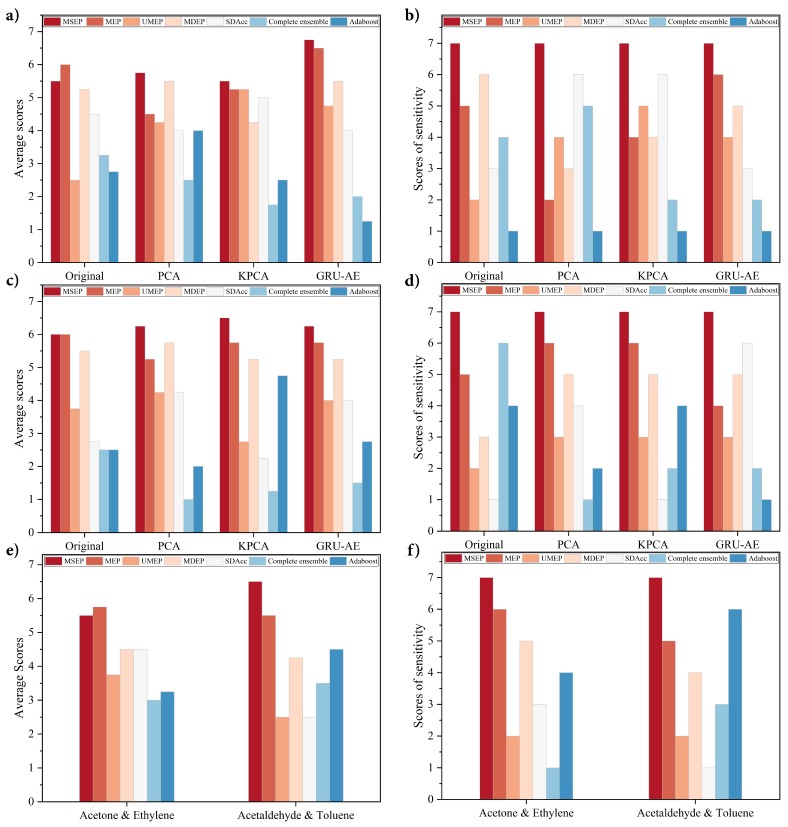
Performance comparison on validation datasets: (**a**) average scores on the Diabetes dataset; (**b**) sensitivity scores on the Diabetes dataset; (**c**) average scores on the gas sensors for home activity monitoring (GSHAM) dataset; (**d**) sensitivity scores on the GSHAM dataset; (**e**) average scores on the gas sensor array drift (GSAD) dataset; and (**f**) sensitivity scores on the GSAD dataset.

Overall evaluation and sensitivity performance of each framework are shown in Figure 4. As shown in Figure 4b,d,f, the proposed MSEP achieved the best sensitivity performance in all the groups. As for average scores, MSEP ranked first in most situations except the original group in Figure 4a and Acetone and Ethylene category in Figure 4e. In these two situations, MEP obtained better average scores than MSEP, but its sensitivity scores were lower than MSEP. The overall performance and sensitivity of UMEP, MDEP, SDAcc, complete ensemble, and adaboost fluctuated greatly in different situations.

## 5. Discussion

This paper presents a novel and reliable GRU-AE-MSEP framework for non-invasive lung cancer detection by the e-nose system. The proposed framework especially contributes to enhancing sensitivity and reducing missed diagnosis rate. The proposed framework was compared with the widely adopted feature extraction methods and existing ordering ensemble pruning techniques. Meanwhile, elaborate ablation experiments based on MSEP and MEP were carried out, which aimed to explore the role of the bonus term in improving sensitivity. To confirm the effectiveness of the proposed framework, all methods were examined under a set of standard metrics, i.e., Acc, Sen, Spe, and AUC. Moreover, all listed methods were experimented on the same dataset collected from patients with different kinds of lung cancer and diverse healthy controls. To further verify the portability of the proposed framework to other signal data, three open source datasets were tested based on the above metrics. In the experiments presented in Section 4.3.1, GRU-AE-MSEP performed best by comparing different feature extractors and classifiers on the collected lung cancer dataset, and the sensitivity achieved by the proposed framework was high and stable. Additionally, the proposed framework had effective classification performance on distinguishing between clinical stages, lung diseases and smoking status. In the experiments presented in Section 4.4, GRU-AE-MSEP was further validated on three open source datasets to test its portability and it outperformed other methods as well.

Dimensionality reduction methods are essential in the analysis of sensor signals, and the extracted principal features perform as a prerequisite for subsequent classification. From the experimental results, metrics of classifiers varied unstably based on PCA and KPCA, while the application of GRU-AE generally improved the performance of classifiers. Since PCA only extracts linear features and cannot deal with nonlinear information, PCA-based frameworks were inferior to those based on original data in several situations. Compared with the original data, the features extracted by KPCA improved the performance of classifiers slightly but were far less effective than the features extracted by GRU-AE. Since conventional feature extraction methods are hand-crafted and require heavy computation as well as domain knowledge, it is hard to judge the impact of the feature extraction process on the final classification results. Moreover, the signal data from e-nose were rather complex, which consisted of linear, nonlinear, and redundant information. As a method based on deep learning training, GRU-AE can process high-dimensional nonlinear data by virtue of automatic feature extraction, especially to process temporal data, which was further verified in Section 4.4.

Ensemble learning is popular in enhancing performance, while ensemble pruning models are developed as efficient improvement techniques by reducing redundant costs in the complete ensemble. Among 56 situations in four datasets, complete ensemble only achieved two highest values in total, i.e., the highest specificity in original lung cancer dataset and in Acetaldehyde and Toluene dataset. It indicated that there existed classifiers with little or negative contribution to the complete ensemble. UMEP, MDEP, and SDAcc are three existing ensemble pruning methods and have been proved to be effective in their original papers. Compared with the complete ensemble and adaboost, experimental results indicate that pruning models were better in the evaluation of overall performance and sensitivity. Therefore, it is reasonable to aggregate classifiers with better performance, and pruning techniques are deemed to be effective for lung cancer non-invasive detection as the results verified.

However, sensitivity and specificity formed a trade-off dilemma when the accuracy was stable and high enough. The aim of calculating average scores was to ensure that the overall performance was not sacrificed as the sensitivity improved. Among seven classification models, the proposed MSEP exhibited more robust performance, which was consistent with the theoretical analysis in Section 3.2.3. By giving up hard samples, UMEP and MDEP were capable of increasing accuracy, but the development space was also limited by hard samples, thus leading to their mediocre performance. The vague and overlapped marking mechanism of SDAcc resulted in fluctuating and unstable ranks in both average scores and sensitivity scores. By adjusting the threshold term, i.e., θ, MSEP can determine what proportion of hard samples to be retained. Instead of abandoning all difficult samples in UMEP and MDEP, the proposed method achieved superb results by taking them into account. Since frameworks based on MSEP achieved the highest average scores and sensitivity scores in every group in lung cancer dataset, MSEP not only improved the sensitivity but also the three other metrics. Therefore, the proposed MSEP can achieve as high sensitivity as possible while ensuring excellent overall performance. In most situations, MEP ranked only second to MSEP in average scores but had unstable ranks in sensitivity in Diabetes and GSHAM datasets, which illustrated the capability of proposed margin-based method in improving classification performance and the effectiveness of bonus term in sensitivity enhancement.

To make the experiments more exhaustive, we investigated as many categories as possible in the experiments presented in Section 4.3.2. For the detection of different clinical stages, stage II had the highest accuracy and sensitivity, which could suggest the valuable prospect of the proposed system for early lung cancer diagnosis. By identifying the COPD and lung cancer, the results were competitive and may provide a further application area. Never versus long-term smokers were distinguished from lung cancer with high accuracy and sensitivity. It may indicate that smoking is a high influence factor for VOC alteration in human breath.

When evaluating on three open source datasets, the performance of the proposed GRU-AE-MSEP framework achieved enormous success as well. MSEP ranked first in every group in terms of sensitivity, and the proposed GRU-AE-MSEP framework obtained the highest accuracy and sensitivity in every dataset, which proved the portability and robustness of the framework. Classification is one of the most popular topics in bioinformatics and disease detection. It is reasonable that one classifier cannot always achieve both highest sensitivity and specificity under certain accuracy, but sensitivity is what we valued and paid attention to. Our practical and transplantable framework demonstrated the ability to promote classification sensitivity in various scenarios.

In the literature, many studies have focused on the detection of lung cancer based on e-nose system, as illustrated in Table 7. The feasibility and effectiveness of the machine learning classifiers were demonstrated on small datasets [66,67,68]. With the development of deep learning methods, the neural network was used by van de Goor [69] and Chang [11]. By contrast, this study provides a new perspective for the non-destructive screening of lung cancer, aiming to design an algorithm to improve the detection sensitivity. In addition to the innovation of feature extraction and classification methods, compared with other studies, the proposed GRU-AE-MSEP framework based on a larger sample size demonstrated superior overall performance and higher sensitivity.

Although the proposed GRU-AE-MSEP framework performed optimally, there is still room for improvement. Primarily, the quantity of the dataset was still limited. To achieve expert-level diagnostic detection, the framework requires more sufficient and diverse data. Secondly, the study of clinical stages, lung diseases, and smoking status is worth delving into in the future. In future research, these limitations in automatic detection of lung cancer could be overcome by using multi-class classification training on gargantuan dataset collected from different types of machine.

## 6. Conclusions

In this paper, a novel intelligent lung cancer diagnosis GRU-AE-MSEP framework is presented. In the process of feature extraction, GRU-AE was introduced to effectively extract principal features from temporal and high-dimensional e-nose signal data. Meanwhile, in the classification process, a heuristic ensemble pruning model was proposed, which enhanced the classification sensitivity while maintaining the overall identification performance. CAD system based on GRU-AE-MSEP is conducive to reducing missed diagnosis of lung cancer and improving survival rate by timely treatment. In the experiment on the collected data, comparative and ablation experiments were conducted under a set of standard metrics to confirm the effectiveness of the proposed framework in lung cancer detection. Additionally, the detection of different stages, diseases, and smokers was implemented to explore the medical application prospect of the proposed framework. Furthermore, three open source datasets were tested, which extended our applicable scenarios and further proved the robustness and adaptability of the framework. Compared with five state-of-the-art classification models and two popular dimensionality reduction methods, the proposed framework achieved superior overall performance with particularly high sensitivity. Therefore, this research can serve as an important step to explore the use of deep learning methods for feature extraction, as well as the use of ensemble pruning techniques for classification in lung cancer diagnosis and other medical detection fields.

## Figures and Tables

**Table 1:** Pattern recognition frameworks for disease diagnosis by electronic nose (e-nose).

First Author	Disease	Samples	Feature Extraction	Classification	Comments
Fens [27]	COPD and Asthma	90	PCA	CDA	The raw data were reduced to four principal components by PCA.
van Velzen [28]	COPD	68	PCA	LR	Breath profiles obtained by GC-MS as well as e-nose proved the non-invasive biomarker for the diagnosis.
Dragonieri [29]	Asthma	40	PCA	LDA	It was the first study in the field of asthma to use pattern analysis to analyze exhaled VOC mixtures collected by e-nose.
Montuschi [30]	Asthma	51	PCA	MLP	The e-nose had high diagnostic performance, but sample size in this study was relatively small for network training.
Liao [31]	VP	140	/	Ensemble NNs	E-nose combined with machine learning algorithms had the advantages of low cost, simple operation and fast response.
Machado [32]	Lung cancer	76	PCA	SVM	The results showed that exhaled gases could distinguish cancer from non-cancer, but had low sensitivity.
Jia [33]	Wound infection	24	KPCA	SVM	Data processed by weighted KPCA had better performance than PCA. The time series data were represented by only seven values, which may cause information loss.
He [34]	Wound infection	80	Handcraft	Autoencoder based self-taught learning	Performance was boosted by self-taught learning based on sparse autoencoder method.

COPD, Chronic Obstructive Pulmonary Disease; VP, Ventilator-associated Pneumonia; PCA, Principal Component Analysis; KPCA, Kernel Principal Component Analysis; CDA, Linear Canonical Discriminant Analysis; LR, Logistic Regression; LDA, Linear Discriminant Analysis; MLP, Multi-layer Perceptron; NN, Neural Network; SVM, Support Vector Machine.

**Table 2:** Demographics of the volunteers in this study.

	Lung Cancer (*n* = 98)		Healthy Controls (*n* = 116)	
Sex	Male	71 (72.4%)	Male	78 (67.2%)
	Female	27 (27.6%)	Female	38 (32.8%)
Age in year (S.D.)		62.8 (6.3)		56.0 (4.0)
Smoking history	Never	35 (35.7%)	Never	46 (39.7%)
	Former	16 (16.3%)	Former	0
	Current	47 (48.0%)	Current	70 (60.3%)
Pulmonary disease	COPD	15 (15.3%)	COPD	34 (29.3%)
	Asthma	2 (2.0%)	Asthma	4 (3.4%)
Histology	Adenocarcinoma	48 (49.0%)		
	Squamous	38 (38.8%)		
	SCLC	12 (12.2%)		
Clinical stages	Stage I	2 (2.0%)		
	Stage II	6 (6.1%)		
	Stage III	44 (44.9%)		
	Stage IV	46 (46.9%)		

S.D., Standard Deviation; SCLC, Small Cell Lung Cancer.

**Table 3:** Results on the lung cancer dataset.

Approach		MSEP		MEP		UMEP		MDEP		SDAcc		Complete Ensemble		Adaboost	
	Metrics	Mean	Std	Mean	Std	Mean	Std	Mean	Std	Mean	Std	Mean	Std	Mean	Std
Original	Acc (%)	**85.00**	5.70	84.27	6.25	82.18	4.71	83.55	5.95	82.36	4.60	76.73	13.72	82.27	5.32
	Sen (%)	**85.87**	6.93	84.76	7.79	80.87	8.65	83.56	6.72	80.70	8.68	67.42	25.76	78.48	8.13
	Spe (%)	84.62	7.84	84.49	7.94	83.58	7.99	84.08	8.33	84.09	7.79	**85.62**	7.44	85.01	7.35
	AUC	**0.83**	0.06	0.83	0.06	0.82	0.05	0.82	0.06	0.82	0.05	0.77	0.12	0.82	0.05
PCA	Acc (%)	**88.09**	7.60	87.09	7.84	83.00	7.32	86.73	7.26	80.82	8.69	75.64	8.52	80.91	8.91
	Sen (%)	**86.43**	12.55	83.57	13.36	79.44	12.04	83.31	11.22	77.90	13.05	74.74	15.53	80.79	10.94
	Spe (%)	90.20	9.18	**91.21**	9.16	85.98	10.02	90.26	9.23	83.67	13.30	77.31	15.17	81.28	12.46
	AUC	0.74	0.04	0.74	0.04	0.73	0.04	0.73	0.04	0.74	0.05	0.66	0.05	**0.81**	0.09
KPCA	Acc (%)	**85.55**	7.26	85.27	7.07	84.36	7.34	84.82	6.66	84.36	7.51	83.55	7.81	83.09	8.08
	Sen (%)	**88.23**	9.52	87.49	10.02	86.33	10.50	87.14	9.91	86.06	10.92	85.36	11.69	85.26	11.87
	Spe (%)	84.45	9.72	**84.60**	9.43	84.11	9.45	84.13	9.22	84.21	9.28	83.09	9.27	82.04	10.29
	AUC	**0.85**	0.06	0.85	0.06	0.84	0.06	0.85	0.06	0.84	0.06	0.84	0.07	0.84	0.08
GRU-AE	Acc (%)	**93.55**	4.90	93.36	4.91	90.55	6.61	91.45	5.35	89.91	6.51	89.82	6.14	91.45	5.03
	Sen (%)	**94.22**	6.44	93.42	6.86	89.91	10.55	90.76	8.86	89.07	10.40	90.89	7.65	91.89	8.31
	Spe (%)	92.80	7.50	**93.11**	7.73	91.09	10.52	91.70	9.98	90.71	9.55	88.54	12.76	91.07	8.92
	AUC	**0.92**	0.05	0.92	0.05	0.91	0.05	0.91	0.05	0.90	0.05	0.90	0.06	0.91	0.05

Best performance is highlighted in bold.

**Table 4:** Results on clinical stages.

Category	Acc (%)	Sen (%)	Spe (%)
Stage II	94.60	97.97	70.00
Stage III	80.80	82.78	81.76
Stage IV	81.00	83.24	81.57

**Table 5:** Results on lung cancer versus pulmonary disease.

Category	Acc (%)	Sen (%)	Spe (%)
Healthy non-smokers	80.00	85.95	76.30
COPD	88.20	92.91	79.60
Lung cancer	85.00	87.23	80.57

Healthy non-smokers: Healthy non-smokers who had no lung disease; Lung cancer: Lung cancer patients who had no other lung diseases.

**Table 6:** Results on smoking history.

Category	Acc (%)	Sen (%)	Spe (%)
Healthy non-smokers	89.60	94.64	84.45
Healthy long-term smokers	92.00	92.42	93.30
Lung cancer	91.20	93.89	88.11

Healthy non-smokers: Healthy non-smokers who had no smoking history; Lung cancer: 15 smokers and 15 non-smokers.

**Table 7:** Comparison of different frameworks in lung cancer detection by e-nose.

Framework	Subjects	Merits	Potential Demerits	Acc (%)	Sen (%)
PCA-CDA [66]	30	Good performance on linear datasets.	Hard to process high-dimensional nonlinear data.	90.00	-
LR [67]	220	a. Simple implementation process.	a. Binary classification must be linearly separable.	81.10	70.00
		b. Consume little computing resources.	b. Hard to handle multi-class features or variables.		
		c. High operation speed.			
RF [68]	143	No feature selection process.	a. Need to integrate all the classifiers.	-	73.30
			b. High calculation cost.		
ANN [69]	167	a. Strong learning ability.	a. Vast parameters exist in the network.	83.00	83.00
		b. Good classification performance.	b. Long training time.		
			c. Insufficient data leads to overfitting.		
			d. Tuning process of ANN is complex.		
PCA-SVM [11]	85	a. Small-sample learning method.	a. Hard to handle nonlinear datasets.		
		b. Rigorous theoretical basis.	b. Hard to implement large-scale training samples.	57.50	63.20
		c. Good robustness.			
PCA-MLP [11]	85	a. Strong learning ability.	a. Hard to handle nonlinear datasets.	75.00	79.00
		b. Good classification performance.	b. Vast parameters exist in the network.		
			c. Long training time.		
			d. Insufficient data leads to overfitting.		
**GRU-AE-MSEP**	**214**	a. Automatic feature extraction process.	a. The tuning process is relatively complex.	**93.55**	**94.22**
		b. Can handle high-dimensional and nonlinear datasets.	b. Training GRU-AE takes a certain amount of time and computing resources.		
		c. Selective ensemble saves testing time and storage.			
		d. High sensitivity and accuracy.			
		e. Less likely to overfit.			

RF (Random Forest); ANN (Artificial Neural Network).

## Appendix A. Parameters Adjustment

**Table A1:** The parameter grid for different methods.

Process	Technique	Adjusted Parameter	Denotation	Range/Set	Step	Default Parameter
Feature Extraction	PCA	Principal components	n	[30, 140]	2	\
	KPCA	Principal components	$n_{k}$	[30, 140]	2	Kernel = rbf Eigen solver = auto
		Sigma	σ	{$10^{1}$, 10${}^{0}$, ⋯, 10${}^{-4}$, 10${}^{-5}$}	\	
	GRU-AE	Units in GRU	\	[30, 140]	2	Batch size = 60
		Layers of GRU	\	[1, 3]	1	Optimizer = adam
		Activation function of GRU	\	{Sigmoid, Relu, Tanh}	\	Learning rate = 0.001
		Epochs	\	[1500, 3000]	100	Decay = $10^{-7}$
Classification	MSEP	Threshold	θ	[−1.0, 0]	0.1	\
	MDEP	Importance balance	α	[0, 1.0]	0.1	\

Considering time, computing resources, and computer performance, only GRUs with three layers or less were adopted.

## Appendix B. Determination of Theta

**Table A2:** Adjusted theta on the collected dataset.

	Original				PCA			
Theta	Acc	Sen	Spe	AUC	Acc	Sen	Spe	AUC
−1.0	84.73	85.71	84.37	0.83	88.00	86.10	90.20	0.74
−0.9	**85.00**	**85.87**	**84.62**	**0.83**	88.00	86.10	90.20	0.74
−0.8	84.73	85.17	84.62	0.83	**88.09**	**86.43**	**90.20**	**0.74**
−0.7	84.82	85.39	84.62	0.83	88.00	85.09	91.29	0.75
−0.6	84.64	85.17	84.50	0.83	87.82	85.29	90.92	0.75
−0.5	84.36	84.73	84.34	0.82	87.55	85.04	90.63	0.74
−0.4	84.18	84.39	84.34	0.82	87.27	84.87	90.06	0.74
−0.3	83.64	83.51	83.99	0.82	86.91	84.51	89.78	0.74
−0.2	83.27	82.62	83.99	0.82	86.00	82.88	89.28	0.74
−0.1	83.09	82.12	83.99	0.82	84.36	81.66	87.25	0.74
0	82.91	82.12	83.70	0.82	81.73	80.51	83.01	0.74
	**KPCA**				**GRU-AE**			
**Theta**	**Acc**	**Sen**	**Spe**	**AUC**	**Acc**	**Sen**	**Spe**	**AUC**
−1.0	85.27	87.31	84.45	0.85	**93.55**	**94.22**	**92.80**	**0.92**
−0.9	85.36	87.73	84.45	0.85	**93.55**	**94.22**	**92.80**	**0.92**
−0.8	**85.55**	**88.23**	**84.45**	**0.85**	93.45	94.22	92.62	0.92
−0.7	85.45	88.03	84.43	0.85	92.91	93.27	92.55	0.92
−0.6	85.27	87.58	84.47	0.85	92.82	92.88	92.70	0.92
−0.5	85.27	87.43	84.63	0.85	92.36	92.09	92.54	0.92
−0.4	85.18	87.66	84.36	0.85	92.45	92.09	92.76	0.92
−0.3	85.27	87.84	84.37	0.85	92.27	91.87	92.62	0.91
−0.2	85.27	87.24	84.87	0.85	91.55	91.01	92.12	0.91
−0.1	84.73	86.80	84.32	0.85	91.09	90.38	91.96	0.91
0	84.36	86.33	84.08	0.84	90.36	90.13	90.63	0.90

Best performance is highlighted in bold.

## Appendix C. Results on Validation Datasets

**Table A3:** Results on the Diabetes dataset.

Approach		MSEP		MEP		UMEP		MDEP		SDAcc		Complete Ensemble		Adaboost	
	Metrics	Mean	Std	Mean	Std	Mean	Std	Mean	Std	Mean	Std	Mean	Std	Mean	Std
Original	Acc (%)	**73.43**	6.21	73.20	7.16	69.89	7.94	71.71	6.72	70.69	8.14	70.69	6.85	68.06	8.87
	Sen (%)	**82.94**	8.17	79.54	9.73	77.61	9.26	79.79	8.50	77.80	10.11	78.37	8.81	74.11	9.13
	Spe (%)	58.27	13.67	**63.77**	13.95	57.58	14.41	59.17	14.49	59.23	14.98	58.48	14.18	57.62	14.62
	Auc	0.63	0.04	0.64	0.04	0.63	0.04	0.63	0.03	0.63	0.04	0.61	0.03	**0.66**	0.10
PCA	Acc (%)	64.69	8.45	63.94	9.01	63.77	8.84	**66.34**	7.17	63.60	9.94	63.14	8.69	61.43	7.76
	Sen (%)	**82.41**	7.03	77.93	8.74	80.37	9.72	80.32	8.19	82.08	11.11	80.65	7.77	68.10	8.76
	Spe (%)	38.50	17.03	43.28	18.60	37.82	14.67	45.27	14.05	35.36	15.12	36.70	15.64	**50.79**	12.37
	Auc	0.57	0.03	0.57	0.04	0.57	0.03	0.57	0.03	0.57	0.03	0.56	0.02	**0.59**	0.08
KPCA	Acc (%)	**73.49**	7.02	72.63	7.12	72.51	6.95	72.40	6.35	72.51	7.20	69.66	7.09	66.40	6.55
	Sen (%)	**82.22**	8.49	79.51	8.81	80.11	8.10	79.51	7.78	80.71	8.25	76.91	9.70	72.28	8.39
	Spe (%)	57.79	14.88	**60.38**	13.47	59.21	14.03	59.66	14.73	57.71	14.37	56.55	14.64	55.54	13.26
	Auc	0.62	0.04	0.62	0.04	0.63	0.05	0.62	0.04	0.63	0.06	0.60	0.05	**0.64**	0.08
GRU-AE	Acc (%)	**82.06**	7.52	81.77	7.64	80.29	8.11	80.91	7.86	79.31	8.03	77.09	8.79	72.46	7.62
	Sen (%)	**85.83**	6.39	84.53	7.18	83.84	9.12	84.11	8.57	82.75	8.49	81.04	9.19	77.46	9.59
	Spe (%)	76.27	13.60	**77.57**	13.87	75.03	15.05	76.18	14.63	73.83	15.67	70.92	15.61	64.77	13.26
	Auc	**0.73**	0.06	0.73	0.06	0.73	0.06	0.73	0.06	0.73	0.06	0.71	0.07	0.71	0.08

Best performance is highlighted in bold.

**Table A4:** Results on the gas sensors for home activity monitoring (GSHAM) dataset.

Approach		MSEP		MEP		UMEP		MDEP		SDAcc		Complete Ensemble		Adaboost	
	Metrics	Mean	Std	Mean	Std	Mean	Std	Mean	Std	Mean	Std	Mean	Std	Mean	Std
Original	Acc (%)	**74.57**	13.79	70.86	18.29	68.57	18.95	70.29	16.12	68.29	19.44	67.71	16.37	65.14	20.63
	Sen (%)	**80.70**	22.46	64.23	33.56	60.07	35.49	63.07	33.68	59.13	35.56	68.90	38.56	64.07	31.21
	Spe (%)	70.10	22.37	81.37	21.23	81.00	19.50	**81.80**	20.78	79.23	22.39	62.90	27.97	69.70	22.80
	AUC	**0.69**	0.10	0.69	0.11	0.68	0.13	0.69	0.12	0.67	0.14	0.59	0.09	0.67	0.20
PCA	Acc (%)	**81.71**	11.44	81.43	11.52	76.00	12.92	80.29	11.39	75.14	18.03	58.86	19.09	66.00	19.56
	Sen (%)	**81.70**	20.53	77.70	21.94	74.30	25.30	76.13	22.02	75.97	25.99	60.37	35.43	66.90	26.94
	Spe (%)	80.83	22.81	83.83	18.74	79.17	20.16	**84.17**	18.65	77.67	24.29	60.33	26.75	67.50	26.94
	AUC	0.72	0.09	0.71	0.09	0.72	0.10	0.72	0.09	**0.74**	0.12	0.66	0.26	0.67	0.19
KPCA	Acc (%)	**80.86**	16.06	80.29	15.62	70.86	16.89	76.29	17.75	70.00	18.57	63.43	20.44	72.29	20.08
	Sen (%)	**87.23**	19.53	82.43	20.85	76.40	26.03	78.33	24.18	74.63	26.74	75.90	26.60	78.17	20.36
	Spe (%)	73.93	28.59	**79.10**	25.72	66.30	26.30	73.87	28.85	66.47	28.36	50.03	30.26	69.47	30.99
	AUC	0.71	0.10	0.70	0.11	0.67	0.13	0.71	0.11	0.67	0.13	0.59	0.10	**0.74**	0.20
GRU-AE	Acc (%)	**89.71**	10.32	88.57	12.45	88.00	14.39	89.43	12.72	85.43	14.43	76.86	20.38	76.00	14.97
	Sen (%)	**92.87**	12.96	87.03	20.49	84.73	23.97	87.90	19.73	88.80	20.08	84.07	22.32	70.43	31.13
	Spe (%)	89.00	15.33	**92.27**	14.75	89.47	22.39	90.03	22.39	83.37	25.03	77.07	28.07	82.90	20.80
	AUC	**0.77**	0.08	0.77	0.08	0.76	0.11	0.76	0.09	0.76	0.10	0.69	0.08	0.77	0.18

Best performance is highlighted in bold.

**Table A5:** Results on the gas sensor array drift (GSAD) dataset.

Category	Metrics Approach	Acc (%)		Sen (%)		Spe (%)		Auc	
		Mean	Std	Mean	Std	Mean	Std	Mean	Std
Acetone and Ethylene	MSEP	99.02	1.55	**98.79**	2.25	99.25	2.45	**0.98**	0.01
	MEP	**99.12**	1.39	98.71	2.28	99.53	1.41	0.98	0.01
	UMEP	98.09	1.59	96.36	2.93	99.88	0.82	0.97	0.01
	MDEP	98.88	1.41	98.31	2.37	99.54	1.38	0.97	0.01
	SDAcc	98.14	1.40	96.43	2.88	**99.92**	0.58	0.97	0.01
	Complete Ensemble	98.00	1.40	96.25	2.79	99.81	0.93	0.97	0.01
	Adaboost	97.63	1.89	96.95	3.00	98.31	2.67	0.98	0.02
Acetaldehyde and Toluene	MSEP	**98.96**	2.23	**98.74**	3.56	99.20	2.42	0.96	0.02
	MEP	98.59	2.44	98.22	3.81	99.09	2.52	0.96	0.02
	UMEP	97.11	3.17	95.75	4.69	98.77	3.25	0.95	0.03
	MDEP	98.15	2.70	97.58	4.04	99.09	2.52	0.95	0.02
	SDAcc	96.44	3.05	94.81	4.78	98.23	3.71	0.96	0.03
	Complete Ensemble	97.56	2.83	95.78	4.74	**99.50**	1.98	0.94	0.02
	Adaboost	97.85	2.68	98.49	3.32	96.60	5.62	**0.98**	0.03

Best performance is highlighted in bold.
